# Thermal Stability and Kinetic Study of Fluvoxamine Stability in Binary Samples with Lactose

**DOI:** 10.15171/apb.2017.006

**Published:** 2017-04-13

**Authors:** Faranak Ghaderi, Mahboob Nemati, Mohammad Reza Siahi-Shadbad, Hadi Valizadeh, Farnaz Monajjemzadeh

**Affiliations:** ^1^Food and Drug Safety Research Center, Tabriz University of Medical Sciences, Tabriz, Iran.; ^2^Department of Drug and Food Control, Urmia University of Medical Sciences, Urmia, Iran.; ^3^Department of Drug and Food Control, Tabriz University of Medical Sciences, Tabriz, Iran.; ^4^Drug Applied Research Center, Tabriz University of Medical Sciences, Tabriz, Iran.; ^5^Department of Pharmaceutics, Tabriz University of Medical Sciences, Tabriz, Iran.

**Keywords:** Fluvoxamine, Lactose, Incompatibility, Kinetic, DSC, Mass

## Abstract

***Purpose:*** In the present study the incompatibility of FLM (fluvoxamine) with lactose in solid state mixtures was investigated. The compatibility was evaluated using different physicochemical methods such as differential scanning calorimetry (DSC), Fourier-transform infrared (FTIR) spectroscopy and mass spectrometry.

***Methods:*** Non-Isothermally stressed physical mixtures were used to calculate the solid–state kinetic parameters. Different thermal models such as Friedman, Flynn–Wall–Ozawa (FWO) and Kissinger–Akahira–Sunose (KAS) were used for the characterization of the drug-excipient interaction.

***Results:*** Overall, the incompatibility of FLM with lactose as a reducing carbohydrate was successfully evaluated and the activation energy of this interaction was calculated.

***Conclusion:*** In this research the lactose and FLM Maillard interaction was proved using physicochemical techniques including DSC and FTIR. It was shown that DSC- based kinetic analysis provides fast and versatile kinetic comparison of Arrhenius activation energies for different pharmaceutical samples.

## Introduaction

Fluvoxamine (FLM) (2-{[(E)-{5-Methoxy-1-[4-(trifluoromethyl) phenyl] pentylidene}amino] oxy} ethanamine) maleate is an *antidepressant drug belonging to* selective serotonin reuptake inhibitor *which is used in* obsessive or compulsive disorders treatment.^1^

Excipients are added in dosage forms to aid manufacture, administration or absorption, appearance enhancement or retention of quality. Excipients may interact with active pharmaceutical ingredients.^2^

Interaction between pharmaceutical ingredients and excipients can affect stability and bioavailability of drugs and consequently influence their safety and efficacy. Thus development of an effective and stable formulation depends on the careful selection of excipients.^2^

A number of physicochemical methods such as Differential Scanning Calorimetry (DSC), Fourier Transform Infrared (FTIR) spectroscopy, Scanning Electron Microscopy (SEM), High Performance Liquid Chromatography (HPLC) and etc. have been used to evaluate the drug- excipient interactions.^3,4^

Since 1970, thermal methods have been used to evaluate the incompatibility of formulation component in pharmaceutical industries.^4-6^

In the pharmaceutical industry, lactose is an appropriate choice of filler due to it has superb compressibility properties. It is also used to form a diluent powder for dry-powder inhalations.^7,8^ Lactose is a reducing disaccharide and can react with amine containing drugs such as FLM during Maillard reaction.^9,10^ The possibility of this chemical reaction lead to conduct this study to provide analytical documentation about the progress of the reaction in solid state pharmaceutical dosage forms and also to study the kinetic of the reaction using non-isothermal DSC techniques.

In this study different analytical methods (DSC, FTIR and Mass spectrometry) were applied to study the FLM- lactose incompatibility reaction and finally the activation energy of the proposed interaction was calculated using different kinetic models.

## Materials and Methods

### Materials

Fluvoxamine maleate (FLM) was purchased from TEMAD Co. (Karaj,Iran). Anhydrous lactose was provided from DMV Chemical Co. (Veghal, Netherlands). All other chemicals were of HPLC grade and were obtained from Labscan Analytical Science (Dublin, Ireland).

### Methods

#### DSC (Differential Scanning Calorimetry)

A DSC-60, Shimadzu differential scanning calorimeter (Kyoto, Japan), with TA-60 software (version 1.51) was used for thermal analysis of FLM and lactose alone, or in binary mixture. Binary mixture (was prepared ( FLM- lactose 1:1 (W/W)), and blended uniformly by tumbling method. Then, DSC pans containing mentioned samples were prepared . and scanned in the temperature range of 25–300°C, with different heating rates (2.5, 10 and 15 °C/min).

#### FTIR (Fourier-transform infrared) spectroscopy

FLM and lactose were blended in 1:1 mass ratios and 20 % (v/w) water was added to each sample according to Serajuddin *et al*. method. and stored in closed vials at 70°C for 72 hours.^11^

FTIR spectra were recorded immediately after mixing and also after storage in oven at specified intervals using potassium bromide disc preparation method (Bomem, MB-100 series, Quebec, Canada).. Processing of FTIR data was performed using GRAMS/32 version 3.04 software (Galactic Industries Corporation, Salem, NH).

#### Mass spectrometry

Mass analysis was performed on the Waters 2695 (Milford, Massachusetts, USA) Quadrupole mass system, at positive electron-spray ionization mode.

## Results and Discussion

### DSC (Differential Scanning Calorimetry)

DSC is widely used in drug-excipient compatibility studies and provides valuable

information such as drug purity ,drug stability, polymorphic forms and their stabilities.^12,13^

**Figure 1:**
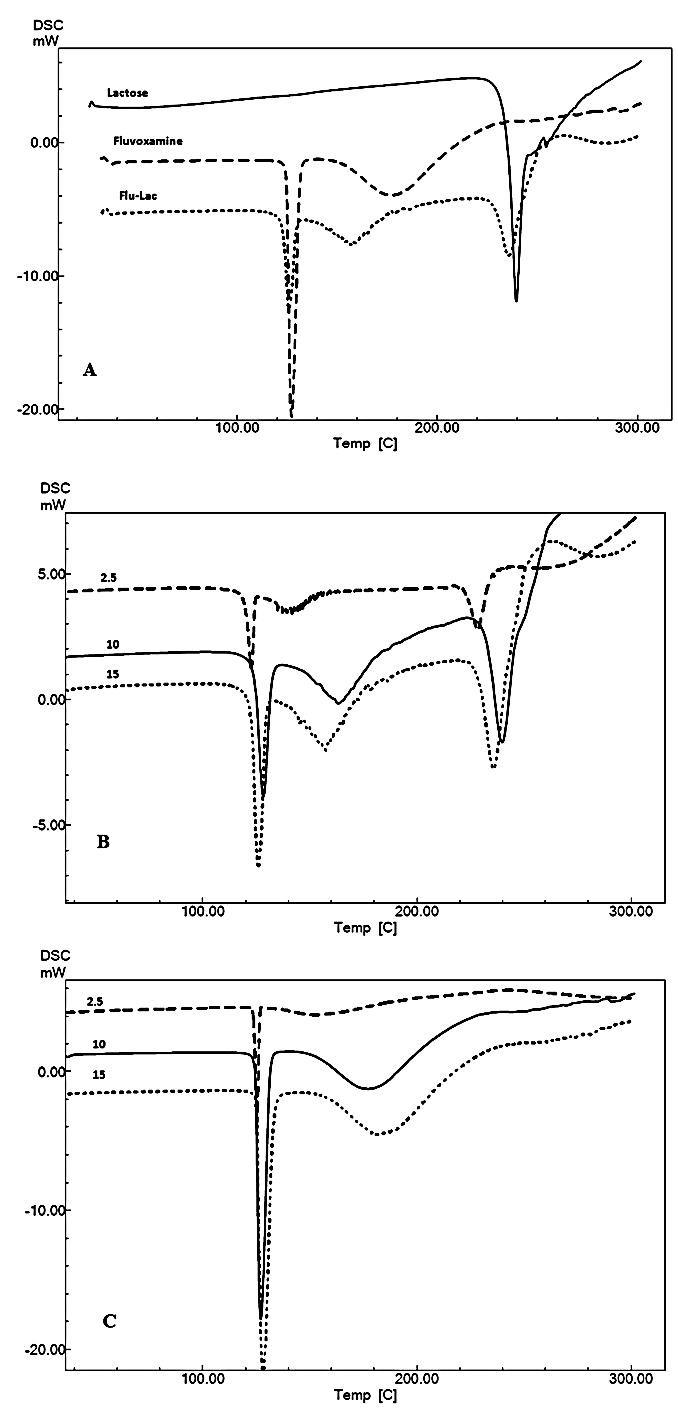
Selected DSC curves of (A) FLM, lactose and FLM-lactose mixture with 1:1 mass ratio (β=10) (B) FLM lactose 1:1 W/W binary mixture at different heating rates (β=2.5, β=10, β=15). (C) FLM at different heating rates (β=2.5, β=10, β=15).

Selected DSC curves of FLM, lactose and FLM - lactose mixture are shown in Figure 1. Thermal behavior of pure drug, pure excipients, and their binary mixture, were analyzed in the DSC curves.

According to Figure 1A, FLM presented its melting point at 127.2°C. The endothermic peak of pure anhydrous lactose appeared at 239.1°C (β=10). This is in accordance with the previous literature.^14^ As shown in the Figure 1A, in FLM-lactose mixture no peak has been added, nor is removed. Therefore simple DSC thermograms at only one heating rate is unable to track the possible Maillard reaction between the drug and excipient and may be misleading for a formulator pharmacist and may result to ignore the incompatibility. As the reaction of type 1 amines with reducing agents is a predictable phenomenon, other DSC based techniques such as multiple scan method at different heating rates and calculation of kinetic parameters for the melting endotherm of the drug substance in the presence and also the absence of the reducing excipient may be useful.

According to Figure 1B and C, while increasing the heating rates, DSC curves were shifted to higher temperatures. It has been previously resulted that the heating rate changes have remarkable influence on the temperature range and the shape of curves.^15^

#### Kinetic study

Recently multiple scan method at different heating rates has gained increasing attention as a fast evaluation method in pharmaceutical preformulation studies.^15^ Friedman (FR), Kissinger–Akahira–Sunose (KAS) and Flynn–Wall–Ozawa (FWO) methods have been extensively applied to study the kinetic parameters in solid state interactions.^16,17^

Equations 1, 2 and 3 are corresponding to the Kissinger–Akahira–Sunose (KAS), Flynn–Wall–Ozawa (FWO) and Friedman methods respectively.

Equation 1 $$\text{ln}\left(\frac{\text{β}}{{\text{T}}^{2}}\right)=\text{ln}\frac{\text{A}\cdot \text{R}}{\text{E}\cdot \text{g}\left(\text{α}\right)}-\frac{\text{E}}{\text{R}\cdot \text{T}}$$

Equation 2 $$\text{lnβ}=\text{ln}\frac{\text{A}\cdot \text{E}}{\text{R}\cdot \text{g}\left(\text{α}\right)}-5.331-1.052\cdot \frac{\text{E}}{\text{R}\cdot \text{T}}$$

Equation 3 $$ln\left(\beta \frac{d\alpha }{dT}\right)=ln\left[A\cdot f\left(\alpha \right)\right]-\left(\frac{E}{R\cdot T}\right)$$

In which, T is the temperature, β is heating rate (°C/min), g (α) is reaction model, E is activation energy, A is the pre-exponential factor, α is the extent of conversion and R is the gas constant.

In KAS method the values of (lnβ/T^2^) were plotted *vs.* 1/T.According to FWO diagram the plot of lnβ vs. (1/T) is linear. The Friedman plot resulted of lnβ∙dαdTvs. (1/T).

**Figure 2:**
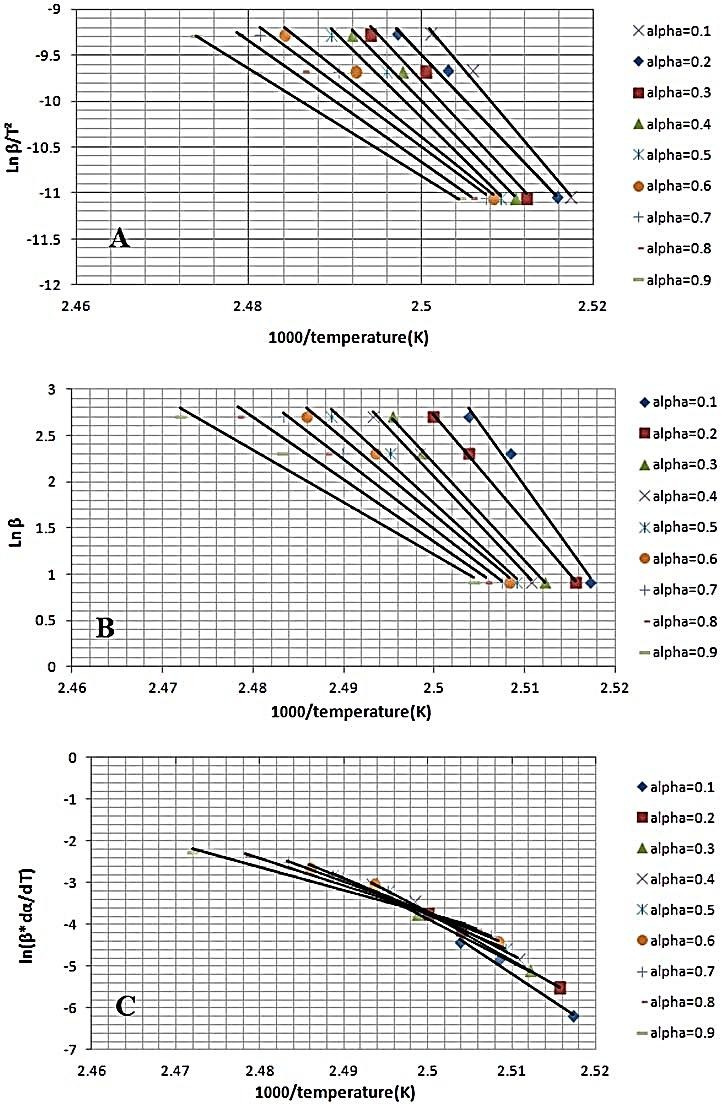
Melting kinetic of pure FLM sample by different models at different heating rates (2.5,10 and 15) and various conversion degrees (α = 0.1 to 0.9). (A) The Kissinger–Akahira–Sunose (B) The Flynn–Wall–Ozawa (FWO) (C) Friedman’s plot.

**Figure 3:**
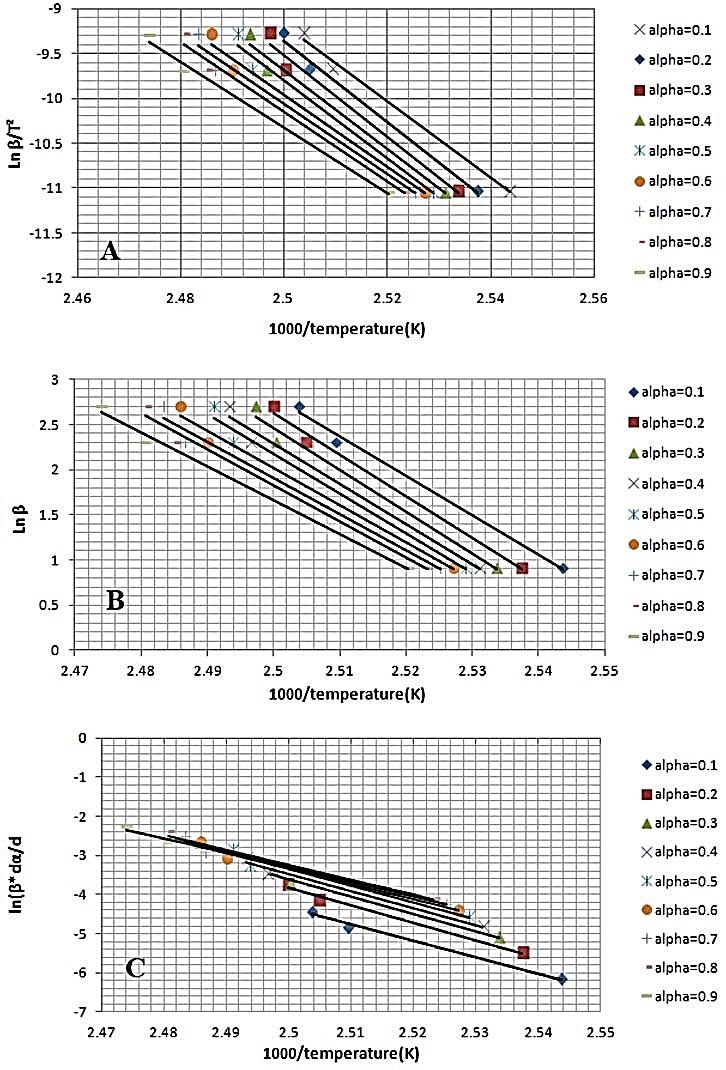
Melting kinetic of FLM in the presence of lactose by different models at different heating rates (2.5,10 and 15) and various conversion degrees (α = 0.1 to 0.9). (A) The Kissinger–Akahira–Sunose (B) The Flynn–Wall–Ozawa (FWO) (C) Friedman’s plot.

In all models, the activation energies (E) of pure FLM and FLM- lactose samples were obtained from slop of the straight lines in Figure 2 and 3 and listed in Table 1 and 2.

**Table 1:** Activation energies calculated for FLM pure sample by the Friedman, Flynn–Wall–Ozawa and Kissinger–Akahira–Sunose methods.

**Method**	**E, (kJ mol–1), for conversion degree, α**									**Mean value**
	**0.1**	**0.2**	**0.3**	**0.4**	**0.5**	**0.6**	**0.7**	**0.8**	**0.9**	
**FR**	1113.39±‏ 9.04	937.03±‏ 9.95	862.81±‏ 6.80	849.45±‏ 6.30	741.14±‏ 15.34	663.84±‏ 6.85	604.32± 13.18	547.06± 4.07	470.6± 15.38	754.41± 4.12
**FWO**	1123.66±‏ 9.42	946.80±11.03	871.06±‏ 9.99	854.07± 13.72	746.39± 7.93	665.75± 9.55	617.05± 9.98	548.97± 1.44	479.08± 12.61	761.50± 3.49
**KAS**	1116.85±‏ 9.69	938.98±‏ 12.71	846.74±9.54	851.38± 9.02	743.06± 12.63	665.75± 9.55	606.22± 15.87	548.95± 1.40	472.48± 12.72	756.49± 4.03

**Table 2:** Activation energies calculated for FLM in the presence of lactose by the Friedman, Flynn–Wall–Ozawa and Kissinger–Akahira–Sunose methods.

**Method**	**E, (kJ mol–1), for conversion degree, α**									**Mean value**
	**0.1**	**0.2**	**0.3**	**0.4**	**0.5**	**0.6**	**0.7**	**0.8**	**0.9**	
FR	352.25±2.47	375.59±6.22	378.62±9.01	366.98 ±11.33	362.74±10.26	338.01±9.88	327.95 ±9.95	325.15 ±6.84	305.96 ±8.53	348.14±2.7
FWO	358.08±4.32	385.88±5.81	387.91±7.19	374.25 ±6.70	372.51 ±10.58	340.95±8.54	338.21 ±9.59	327.58 ±4.82	315.70 ±7.48	355.78±2.09
KAS	352.26±3.20	377.58±3.41	380.60±6.21	368.94 ±8.55	367.20 ±11.03	339.95±7.13	332.36 ±10.75	327.08 ±4.11	309.38 ±7.94	350.60±2.95

As shown in Tables 1 and 2 the results obtained by mentioned kinetic methods are in a good agreement (P value > 0.05) and small standard deviation values showed the acceptable reproducibility.Also the mean activation energy calculated using these methods for the pure FLM is about 2-fold higher than that of FLM - lactose mixture. This can be explained by the fact that, FLM is thermally more‏ stable than its mixture with lactose which can be due to their incompatibility reaction.

In a study Fulias et al. evaluated thermal decomposition of pure cefadroxil and its mixture with excipients using TG/ DTG and DSC techniques and presented their activation energies. Based on their results the calculated activation energy for cefadroxil was too higher than that of cefadroxil and magnesium stearate binary mixtures, thus the incompatibility was concluded and reported accordingly.^18^

#### FTIR (Fourier-transform infrared) spectroscopy

**Figure 4:**
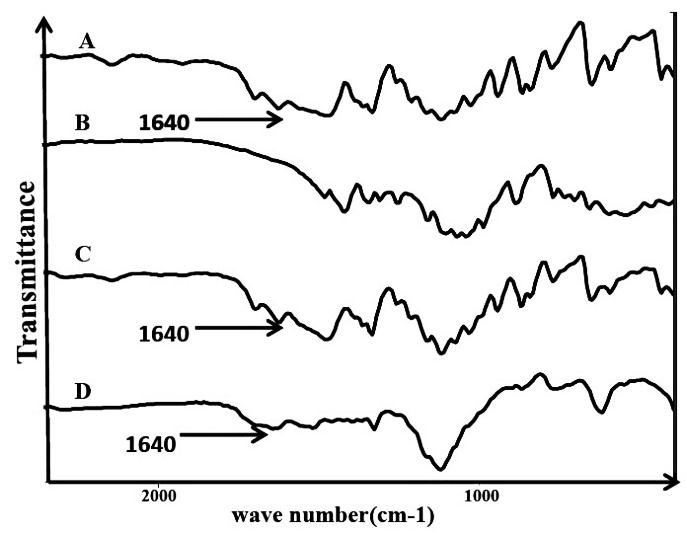
TIR spectra of (A) FLM (B) lactose (C) FLM -lactose 1:1 W/W binary mixture immediately after mixing, and (D) binary mixture with 20% added water after 72 hours incubation at 70 °C.

IR spectra of FLM, lactose and, FLM-lactose mixture immediately after mixing, and 72 hour after incubation in oven (t=70°C) are shown in Figure 4.

FLM IR’s main signals appeared at ~ 1698 cm^-1^(C=N stretching vibration), 1624 cm^-1^(primary amines N–H bending), 1474 cm^-1^(CH_2_ symmetric deformation vibration in O―CH2―), 1336, 1162 and1117 cm^-1^(general range for C-F stretching vibration), 839 and 866 cm^-1^(out-of-plane deformation vibrations R―Ar―R).

Lactose– FLM mixture’s main signals were corresponding to the component’s Peaks. It was

shown that N–H bending vibration at about 1624‏ which is a specific absorption for primary

amines showed a significant decrease in drug-excipient mixture after 72 hours storage in 70°C .

This can be indicative of a drug- excipient interaction.

#### Mass spectrometry

Condensation products of lactose and different drug substances such as hydrochlorothiazide, fluoxetine and metoclopramide have detected in several investigations.^9,19,20^ We have previously studied the compatibility of acyclovir, baclofen, gabapentin and doxepin with lactose and dextrose in physical mixtures and commercial tablets using mass spectrometry^21-25^ Based on the mass results in this study the condensation product of FLM with lactose was successfully detected.

**Figure 5:**
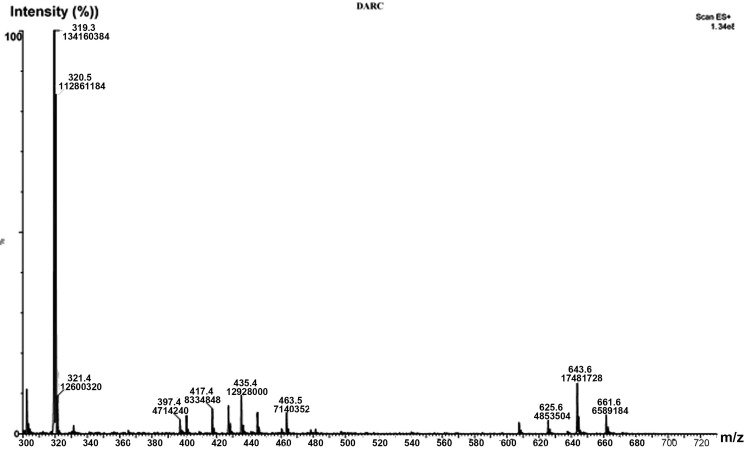
Positive ion mode electrospray mass spectrum of sertraline-lactose mixture after 5 hours storage at 90°C

Physical mixture of FLM and lactose with 20% added water was stored at 80 °C for 24 hours. This sample was injected to the mass system. Mass spectra are presented in Figure 5. The full-scan positive ion electrospray product ion mass spectra showed that the molecular ion of FLM was the protonated molecules [M+H]^+^ of m/z 319.0. This is accordance with the previous reports.^26^

Proposed structures for Maillard type interaction products have been presented in Figure 4. The m/z value at 643.6 is related to [M+H] ^+^ of compound 1 in Figure 4 which can be converted to compound 2 during proton transfer reaction. Open ring of the saccharide moiety in compound 2 may be closed to Pyranose and Furanose forms, producing compound 3 and 4 with the same molecular mass. According to the previous studies the Maillard reaction finally leads to the formation of N-Formyl compound (compound 5). In the current evaluation there was no documentation about the formation of this end stage product between FLM and lactose. This can be attributed to the incomplete reaction progress in the defined conditions of this study.

## Conclusion

DSC, FTIR and mass spectrometry were used to detect FLM- lactose incompatibility. Although simple DSC was not successful to track the incompatibility but multiple scan at different heating rates resulted in a higher activation energies for pure drug compared to its binary mixture with lactose which can be indicative of the incompatibility. It should be kept in mind that sometimes simple DSC curves are unable to report the incompatibility and may be misleading. Thus other techniques should be used to evaluate the stability of the drug in the samples. In this study FTIR and subsequently mass analysis proved the Maillard type incompatibility between FLM and lactose.

The safety of Maillard reaction products were studied several investigations and their genotoxic, carcinogenic, or cytotoxic potential has been examined in food products.^27^ There have been no safety evaluation performed in the pharmaceutical field until now but it is recommended that avoiding the combination of FLM with lactose in pharmaceutical formulations may have different benefits such as decreased potential of drug loss due to unwanted drug-excipient interaction and also increased safety issues.

## Acknowledgments

This paper was extracted from a PhD thesis (No: 91) submitted to faculty of Pharmacy, Tabriz University of Medical Sciences and financially supported by the same University.

## Ethical Issues

Not applicable.

## Conflict of Interest

The authors declare no conflict of interests.

**Figure 6:**
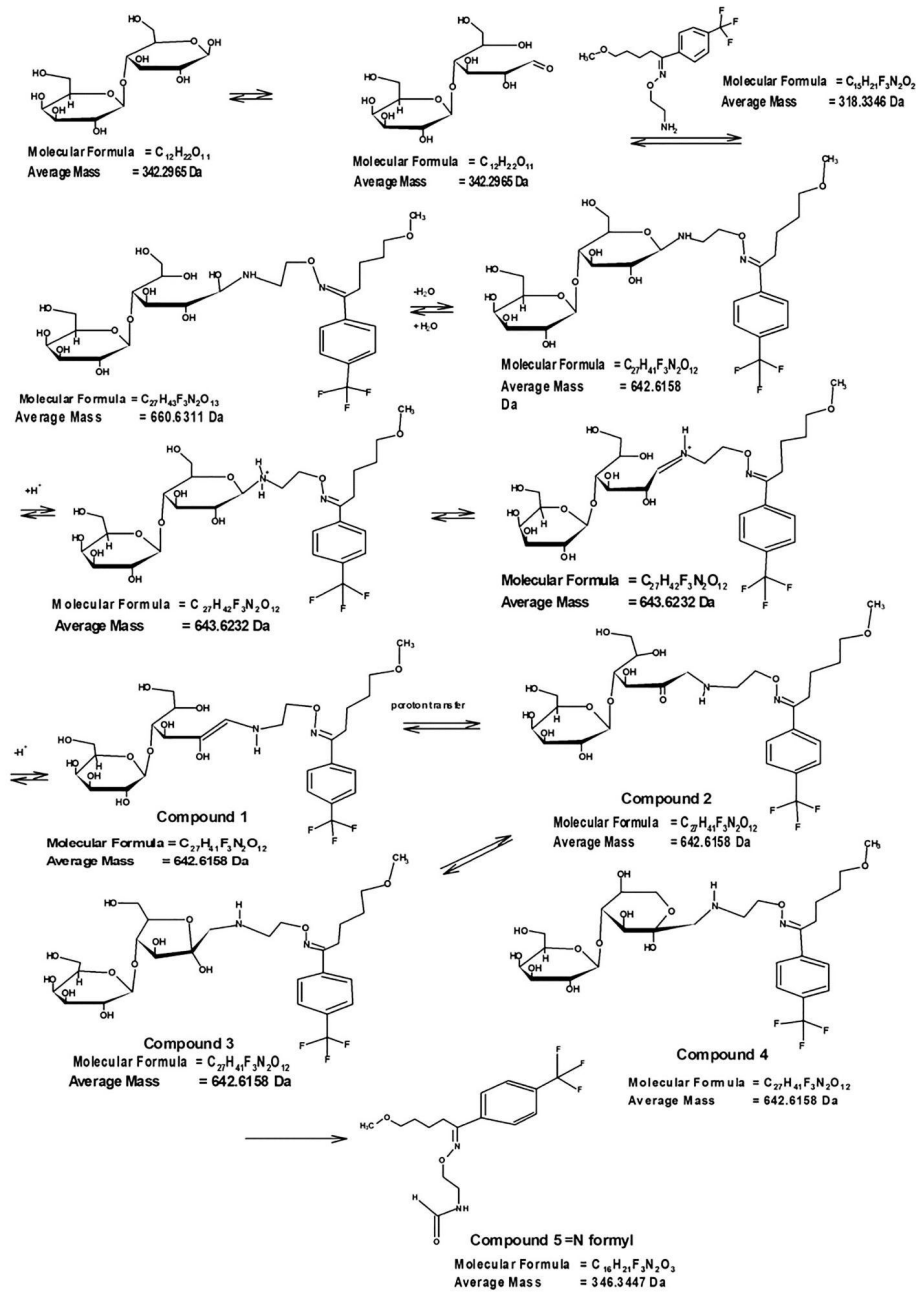
Proposed structures for Maillard reaction of FLM with lactose.
